# Mapping action naming in patients with gliomas: The influence of transitivity

**DOI:** 10.1016/j.ynirp.2023.100184

**Published:** 2023-09-21

**Authors:** Effrosyni Ntemou, Klara Reisch, Frank Burchert, Roel Jonkers, Thomas Picht, Adrià Rofes

**Affiliations:** a Center for Language and Cognition (CLCG), University of Groningen, Groningen, the Netherlands; b Department of Neurosurgery, Charité – Universitätsmedizin Berlin, Berlin, Germany; c International Doctorate for Experimental Approaches to Language and Brain (IDEALAB), University of Groningen (NL), University of Potsdam (DE), Newcastle University (UK), Macquarie University (AU), Australia; d Department of Linguistics, University of Potsdam, Potsdam, Germany; e Cluster of Excellence: “Matters of Activity. Image Space Material”, Humboldt University, Berlin, Germany

**Keywords:** nTMS, Language mapping, Glioma, Presurgical mapping, Language, NBS

## Abstract

**Objective:**

Patients with left perisylvian gliomas might undergo language mapping with nTMS in preparation for awake brain surgery. Action naming is an important addition to the presurgical language mapping protocol. However, it has not yet been determined whether specific action stimuli can influence mapping outcomes in terms of number and/or localisation of induced errors.

**Methods:**

We investigated this question by employing tractography-based nTMS language mapping of the left arcuate fasciculus (AF) with two types of verbs: transitive and intransitive. Data were collected from 22 patients with a left perisylvian glioma.

**Results:**

Our results demonstrated that nTMS induced a higher error rate with transitive rather than intransitive verbs, specifically during stimulation of the posterior temporal terminations of the left AF (transitive error rate: 8.3%; intransitive error rate: 4.8%). The effect was absent when gliomas displaced the temporal terminations of the AF. Also, nTMS triggered a higher number of semantic errors with transitive (vs intransitive) actions during stimulation of the posterior temporal terminations of the AF (semantic error rate – transitives: 3.3%; semantic error rate – intransitives: 0%).

**Conclusion:**

Our work highlights that clinical outcomes of language mapping with nTMS are affected by the choice of linguistic stimuli. Transitive verbs may be suited to achieve optimal nTMS mapping outcomes in posterior temporal areas of the left AF in this population. Displacement of white matter terminations due to the tumor can affect these results, and semantic errors may indicate core language processes that can be mapped when administering transitive verbs.

## Abbreviations

nTMS: navigated Transcranial Magnetic Stimulation
AF: Arcuate Fasciculus
DES: Direct Electrical Stimulation
DTI: Diffusion Tensor Imaging
SMG: Supramarginal Gyrus
STG: Superior Temporal Gyrus
MTG: Middle Temporal Gyrus
ITG: Inferior Temporal Gyrus
IFG: Inferior Frontal Gyrus
fMRI: functional Magnetic Resonance Imaging
WHO: World Health Organization
NBS: Navigated Brain Stimulation

## 1 Introduction

Stimuli with different linguistic characteristics may be processed in varying regions in the brain, also impacting clinical language mappings (Rofes and Mahon, 2021). In this paper, we aim to deepen our understanding of the linguistic variables that affect presurgical language mapping with nTMS in people with gliomas in the left-dominant language hemisphere. We focus on two major stimuli of action naming: transitive and intransitive verbs. Specifically, we ask whether more nTMS errors will be identified with transitive rather than intransitive verbs, when stimulating specific terminations of the AF and/or peritumoral regions. Given that tractography-based nTMS is increasingly used as a presurgical language mapping method (Reisch et al., 2022), answering these questions will enable clinicians to make informed decisions regarding which items should be used for optimal presurgical mapping outcomes. The results may also be extended to intraoperative DES mappings.

Verbs can differ in terms of the number and type of roles their meanings require (i.e., *thematic roles*). Some verbs, for instance, can only express actions that involve one entity or role (e.g., *to cough* as in *John is coughing*), whereas other verbs can express actions that involve more than one role (e.g., *to cook* as in *John is cooking a fish*; Grimshaw, 1990). The intransitive verb *to cough* takes up one entity with the thematic role of *agent* (i.e., the entity executing the action, *John*). The transitive verb *to cook* takes up two entities: an *agent* (i.e., the entity executing the action, John) and a *theme* (i.e., an object undergoing motion or change of state, *a fish*). These semantic differences of verbs requiring one or more thematic roles depending on their meaning also translate to syntactic differences (Grimshaw, 1990; Rappaport Hovav and Levin, 2002). For example, in German, objects that have the role of the *theme* are marked with accusative case (e.g., *John is cooking a fish - John kocht eine****n* Fisch**_theme-accusative_).

Evidence on how the brain processes transitive and intransitive verbs traditionally comes from lesion and neuroimaging studies. Patients with post-stroke aphasia use on average fewer transitive and more intransitive verbs than healthy controls during spontaneous speech (Bastiaanse and Jonkers, 1998). Similarly, they have more difficulties naming transitive than intransitive verbs (De Bleser and Kauschke, 2003). During fMRI healthy adults exhibit more BOLD activation in left perisylvian regions. Specifically, in inferior parietal and posterior temporal regions, such as the SMG and AnG (Malyutina & den Ouden, 2017; Shetreet et al., 2007). One study that assessed language production reported additional increased activity in the left IFG (den Ouden et al., 2009). In people after stroke, damage to the left posterior temporal cortex as well as the AnG affected verbs with more thematic roles using VLSM (Ouden et al., 2019).

Recent work with neurostimulation techniques has been used to indicate that tDCS over the left IFG rather than posterior temporal cortex affects judgments on whether actions are transitive or intransitive (den Ouden and Zhu, 2022). DES work on action and object naming demonstrates that separate brain regions can be identified as language-relevant depending on the task used (Rofes et al., 2017; Rofes and Miceli, 2014). Such findings suggest that the left SMG and IFG are particularly involved in the processing of actions rather than objects (Corina et al., 2010). Within the preoperative language mapping setting using nTMS, similar results have been reported, albeit inconsistently (Hernandez-Pavon et al., 2014; Ohlerth et al., 2021). Importantly, specific to our question, nTMS over the left parietal lobe affects naming of transitive actions (Ntemou et al., 2021), and stimulation of the left intra-parietal sulcus affects both the production and comprehension of transitive actions (Finocchiaro et al., 2015; Vercesi et al., 2020; Ward et al., 2022).

In summary, previous work demonstrates that transitive and intransitive verbs may differently involve perisylvian areas of the left hemisphere. These regions can be probed with neurostimulation techniques, such as nTMS, in the preoperative language mapping setting to understand whether further errors can be identified with transitive vs intransitive verbs. Answering this question could potentially maximize the sensitivity and specificity of pre- and intraoperative stimulation protocols. The results may be additionally enhanced when TMS is directed to cortical terminations of association tracts (Reisch et al., 2022).

## 2 Methods

### 2.1 Participants

**Fig. 1:**
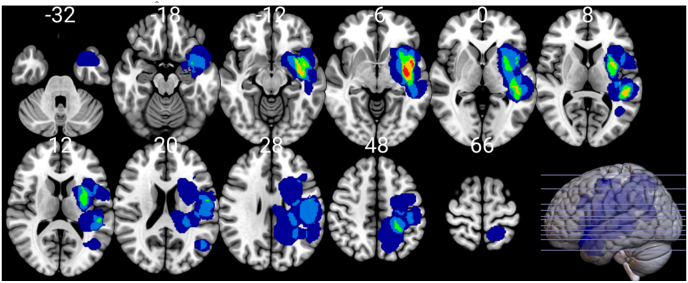
Lesion overlay for patient sample. Warmer colors indicate higher number of patients with given voxel lesioned. Numbers above axial slices indicate that axial slice position in MNI space. Scan orientation follows the radiological convention. (For interpretation of the references to color in this figure legend, the reader is referred to the Web version of this article.)

Twenty-two individuals with gliomas participated in the study (mean age = 49.5, SD = 14.9, female = 9). All participants were native German speakers and right-handed according to the German version of the Edinburgh Handedness Inventory (Oldfield, 1971). Participants had gliomas in the left hemisphere with the majority having a WHO grade IV glioma (II = 27.3, III = 13.7, IV = 59%). Tumors were located in the left perisylvian regions, and their overlay is shown in Fig. 1. Additional inclusion criteria were: (1) age of at least 18 years, (2) no contra-indication for Magnetic Resonance Imaging (MRI) and nTMS mapping (e.g., cardiac pacemakers, deep brain stimulation devices, cochlear implants), and (4) no diagnosis of other neurological or psychiatric disorders. The study was approved by the local ethics committee of the hospital and was performed according to the Declaration of Helsinki. All individuals signed an informed consent prior to the nTMS session.

### 2.2 MRI data acquisition

MRI images were acquired using a Siemens Skyra 3T scanner (Erlangen, Germany) equipped with a 32-channel receiver head coil. The scans consisted of T1-weighted images (TR/TE/TI 2300/2.32/900 m s, 9° flip angle, 256 × 256 matrix, 1 mm isotropic voxels, 192 slices, acquisition time: 5 min) and diffusion MRI single-shell data (TR/TE 7500/95 m s, 2 × 2 × 2 mm 3 voxels, 128 × 128 matrix, 60 slices, acquisition time: 7 min). Structural scans were then uploaded to the server-based clinical planning software iPLAN NET 3.0 (Brainlab AG, Feldkirchen, Germany) and were denoised as well as corrected for eddy-currents, Gibbs ringing artifacts, and subject movement.

### 2.3 Tractography

**Fig. 2:**
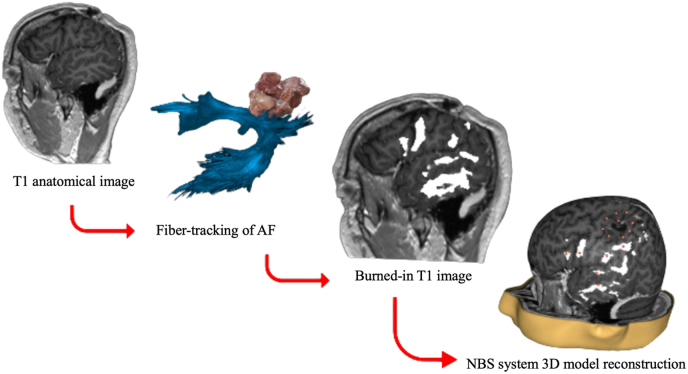
Visualization of methodological steps prior to nTMS mapping. After the left AF is tracked using iPLAN NET 3.0 (T1 anatomical image + Fiber-tracking of AF), the generated object of the tract is burned into the T1 (Burned-in T1 image). The burned-in T1 image is then used to place AF and peritumoral stimulation targets (NBS system 3D model reconstruction).

Tractography of the left AF was performed using deterministic algorithms (FACT and TEND) on iPLAN NET 3.0 (Brainlab, Feldkirchen, Germany). Fiber tracking of the AF was performed manually using a 2 ROI approach following previous clinical protocols (Catani and de Schotten, 2008; Fekonja et al., 2019). The seed ROI was placed in the white matter region underneath the central sulcus and the second ROI was placed more laterally at the level of the posterior superior temporal gyrus and the temporoparietal junction (Fekonja et al., 2019; Reisch et al., 2022). Our posterior ROI targeted terminations of AF sections in posterior temporal and inferior parietal regions. Following previous methodologies for language-relevant tractography for patients with gliomas, if the tumors infiltrated the anatomical landmarks used for ROI placement, subject-individual ROIs were used (Tuncer et al., 2021). Following standard thresholds, fractional anisotropy was set at 0.15, minimum length at 50 mm, and maximum angulation at 30°. In cases when spurious streamlines were detected due the location of the tumor or oedema, exclusion ROIs were additionally used. The AF pathway was then converted to a binary object and was burned-in to the anatomical T1-weighted image using the “Burned-in export” functionality offered by Brainlab Elements. The steps of the process are visualized in Fig. 2.

### 2.4 nTMS stimulation targets & coordinate normalization

Following Reisch et al. (2022), the burned-in anatomical T1-weighted image was then uploaded to the nTMS software (Nexstim eXimia NBS, system version 4.3) to guide the placement of stimulation targets and reconstruct the 3D representation of each participant's brain. The peeling depth of the 3D brain was set at 21.5 mm. Two stimulation targets were placed over the terminations of the left AF in the inferior frontal gyrus, two more over the cortical terminations of the tract in the posterior temporal lobe and two more over the termination in the inferior parietal lobe. Stimulation point placement was guided by the individual AF tractograms and previous literature of cortical regions associated with the processing of transitive compared to intransitive verbs (see 1. Introduction; stimulation targets in MNI space are given in Supplementary Material, Table S3). Additionally, ten peritumoral stimulation targets were set over the borders of the tumor. The AF stimulation targets were stimulated 6 times each, whereas each peritumoral point was stimulated 10 times. For one patient, stimulation of AF terminations was not possible due to technical issues. For another 5 patients, some peritumoral targets were not stimulated due to technical issues or induced pain (see Supplementary Material, Table S3).

The individual anatomical coordinates of all stimulation points were collected and registered to Montreal Neurological Institute 152 (MNI) space using FSL (https://fsl.fmrib.ox.ac.uk/fsl/). To do this, we first skull-stripped the individual T1-weighted images using *optiBET* and then co-registered the skull-stripped image using both lineal (*FLIRT*) and non-linear (*FNIRT*) registration to MNI space (Jenkinson et al., 2012; Lutkenhoff et al., 2014; Smith, 2002). After successful registration of the anatomical T1-weighted image, we converted the individual coordinates to MNI coordinates using the matrix generated during co-registration as well as the FSL command *img2imgcoord*. To localize the cortical location of the stimulation targets, we used the Harvard-Oxford cortical atlas (Makris et al., 2006) and the command *atlasquery* on FSL.

### 2.5 Materials & procedure

**Fig. 3:**
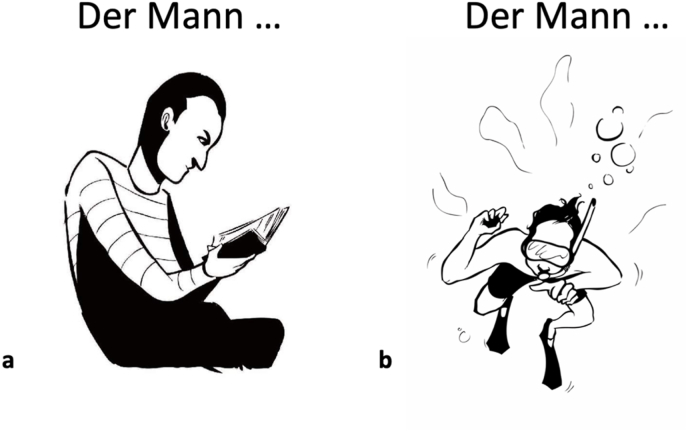
Examples of a transitive verb (a) that elicits the sentence *“Der Mann liest/The man reads (a book)”* and an intransitive item (b) that elicits the sentence *“Der Mann taucht/The man snorkels”*.

The action naming task used during language mapping contains overall 75 items (Ohlerth et al., 2020). One item did not perform well during the piloting phase of the present study and was therefore excluded from the stack. This resulted in overall 74 actions, 22 of which were intransitive and 52 were transitive actions. The images provided the subject of the sentence through a lead-in phrase *“Der Mann../Die Frau …” (“The man … /The woman …”).* Underneath this phrase, a pictogram of the elicited action was displayed. Participants were instructed to produce the lead-in phrase with the verb inflected for person, number, and tense. During the naming of transitive actions, participants were not requested to produce the object of the action. This did not affect the grammatical correctness of the produced sentences (e.g., *“Der Mann ließt (eine Zeitung)”; “The man reads (a newspaper)”*). Transitive and intransitive item lists did not differ in terms of frequency, age of acquisition and naming accuracy (see Supplementary Material – Table S4). Examples of a transitive and an intransitive item are shown in Fig. 3.

For language mapping, a figure-of-eight coil was used for repetitive nTMS. We measured the resting motor threshold (RMT) of each individual for the first dorsal interosseus muscle (FDI) of the right hand, following published guidelines for presurgical language mapping (Krieg et al., 2017). As is typically the case for presurgical language mapping, we performed repetitive stimulation with frequency set at 5 Hz/5 pulses (Krieg et al., 2017). The stimulation intensity for language mapping was then set at 110% of each participant's ipsilateral RMT. Baseline testing was performed twice without any stimulation. Items that were named incorrectly were excluded from naming under stimulation. Picture presentation was set at 1200ms and inter-picture interval at 2500ms (Krieg et al., 2017). Presentation of images was randomized for each participant. Duration of nTMS language mapping did not exceed one and a half hours and was part of the clinical workup in preparation for awake surgery. All mappings were performed within one week prior to the surgery.

### 2.6 Error classification

Errors induced by nTMS were classified into 8 categories, according to previous literature (Picht et al., 2013; Rofes et al., 2017): (1) No response: Complete lack of response; (2). Anomia: Production of intelligible lead-in phrase but missing target action (e.g., *Der Mann …; The man …*); (3) Hesitation on the target: Production of the lead-in phrase but delayed production of the target action; (4) Hesitation on the sentence: Delayed production of the entire sentence, including the lead-in phrase; (5) Semantic paraphasia: The target action is replaced by an existent but different word (e.g., *Der Mann isst; The man eats* for the target *Der Mann kocht; The man cooks*); (6) Grammatical error: Missing or incorrect inflection of the verb (e.g., *Der Mann koch; The man cook* for the target *Der Mann kocht; The man cook****s***); (7) Phonological paraphasia: Phonemes are substituted or missing for the target word (e.g., *Der Mann rocht; The man rooks* for the target *Der Mann kocht; The man cooks*); (8) Articulation error: Stuttered of slurred speech (e.g., *Der Mann k-k-kocht; The man c-c-cooks* for the target *Der Mann kocht; The man cooks*).

### 2.7 Statistical analysis

We conducted analyses considering the sum of errors during nTMS to terminations of the AF and peritumoral regions, as well as only during nTMS to terminations of the AF and only to peritumoral regions. We used a mixed effects logistic regression to assess whether more nTMS errors occurred during the production of transitive rather than intransitive items. To check whether number of errors for the different terminations varied, we calculated error rates for each verb type according to AF terminations in the 3 different lobes (frontal, temporal, parietal). We conducted Barnard's tests to examine whether numbers of nTMS errors between transitive and intransitive actions differed for terminations in peritumoral regions (e.g., posterior SMG). Barnard's tests were also used to investigate whether different error types were affected by stimulation according to action type (*transitive* vs *intransitive*) and stimulated region. All analyses were conducted using R (R Core Team, 2020). Logistic mixed models were calculated using the *lme4* package (Bates et al., 2014) and Barnard's tests using the *Barnard* package (Erguler, 2016). Barnard's tests have been reported to be more powerful for 2 × 2 tables of low binary outcomes compared to Fisher's exact tests (Barnard, 1947; Suissa and Shuster, 1985). Additional analyses were conducted to cross-validate the results.

## 3 Results

### 3.1 Baseline errors

During two rounds of baseline naming the overall error rate was 30.7%. Excluded intransitive items were on average 25.7% (n = 124), whereas excluded transitive items were 33.2% over both baseline rounds (n = 380). Although baseline error rates were above 25%, only effortlessly and correctly named items were included in the stimulation rounds. Additionally, a non-parametric *t*-test revealed that this difference was not statistically significant (t = 2.0, p > 0.05). Additional chi-square tests for each participant showed that no patient was significantly impaired in producing transitive over intransitive verbs during both rounds of baseline naming (see Supplementary Material; Table S2).

### 3.2 AF terminations + peritumoral regions

**Fig. 4:**
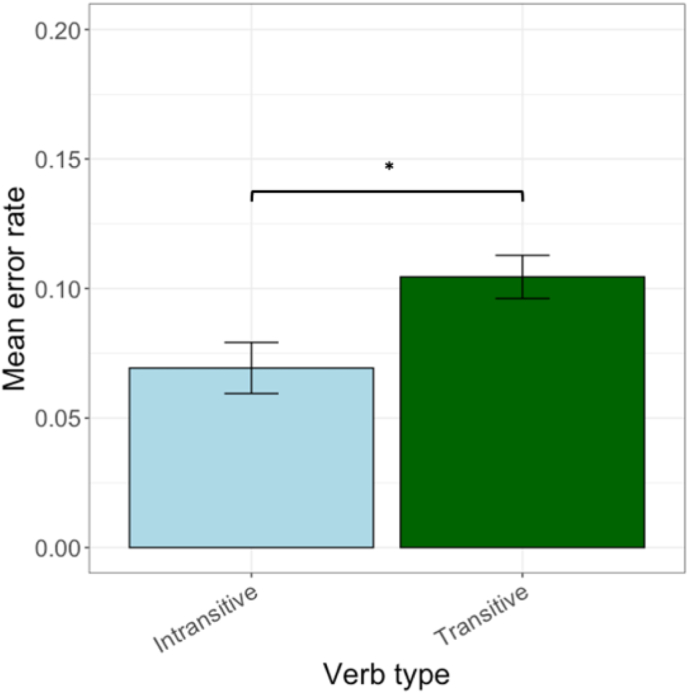
Overall nTMS-induced error rate for the two verb types (*: p < 0.05). Error bars display the standard error (SE).

The total number of stimulations across AF terminations and peritumoral regions was 3106 with 280 stimulations inducing language errors (9.02% overall error rate). Out of the 2051 stimulations with transitive items, 209 resulted in nTMS errors (10.02%). Out of the 1055 stimulations with intransitive items, 71 resulted in nTMS errors (6.7%). To test whether the difference between nTMS errors for transitive and intransitive verbs differed, we conducted mixed effects logistic regression models. An ANOVA between the different models demonstrated that the biggest amount of variance (AUC = 0.73; AIC = 1802) in our data was explained by the model that included random intercepts for items and participants (i.e., *Error binary ∼ Verb type* + *(1|Item)* + *(1|Participant))*. The results of the model demonstrated that nTMS induced more errors during transitive rather than intransitive action naming (β = 0.59, p = 0.025). Fig. 4 shows the mean error rates for each verb type and results of the logistic model can be found in Supplementary Material (Table S1).

### 3.3 AF terminations

**Fig. 5:**
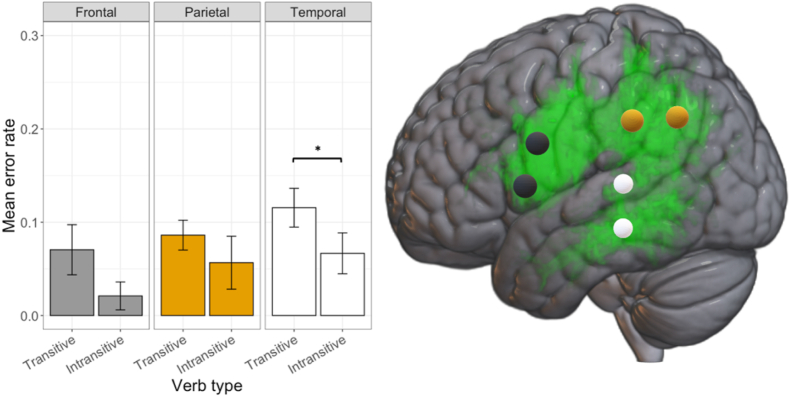
Error rates according to termination of the left AF and verb type alongside exemplary image for stimulation target placement (grey: frontal terminations; yellow: parietal terminations; white: temporal terminations; *: p < 0.05). Error bars display the standard error (SE). (For interpretation of the references to color in this figure legend, the reader is referred to the Web version of this article.)

Approximately 8.5% of all stimulations that were directed at the cortical terminations of the AF resulted in nTMS errors (998 stimulations; 85 errors). 22.4% of total nTMS errors were induced over the frontal terminations, 37.6% over the temporal, and 40% over the parietal terminations of the left AF. Regarding error induction differences between the two verb types, nTMS induced 8% of errors during transitive verb naming and 2.8% with intransitive verb naming over the frontal terminations. Over the temporal terminations of the left AF, the rate of nTMS errors for transitive verbs was 8.3% whereas the intransitive error rate was 4.8%. During transitive verb naming the nTMS error rate was 10.7% and intransitive 7% when the parietal terminations of the AF where stimulated. Barnard's tests conducted for each termination showed that only over the temporal terminations of the left AF, nTMS induced a significantly higher error rate during transitive verb compared to intransitive verb naming (W = −2.52, p = 0.01; Fig. 5).

### 3.4 Peritumoral regions

The nTMS error rate of peritumoral regions was 9.2% (2108 total peritumoral stimulations). Across all peritumoral stimulations, a logistic regression model showed that nTMS induced significantly more errors during transitive compared to intransitive verb naming (10.1% and 7.4% respectively; β = 0.33, p = 0.04). Looking at the specific peritumoral areas that were affected according to the Harvard-Oxford cortical atlas, Barnard's tests indicated that nTMS over the posterior SMG induced significantly more errors during transitive compared to intransitive verb naming (W = 2.05, p = 0.03). In areas such as the posterior STG, anterior SMG, and the ventral PrG error rates between the two verb types approached significance, without however crossing the α = 0.05 level.

### 3.5 Cross-validation analysis: temporal terminations

**Fig. 6:**
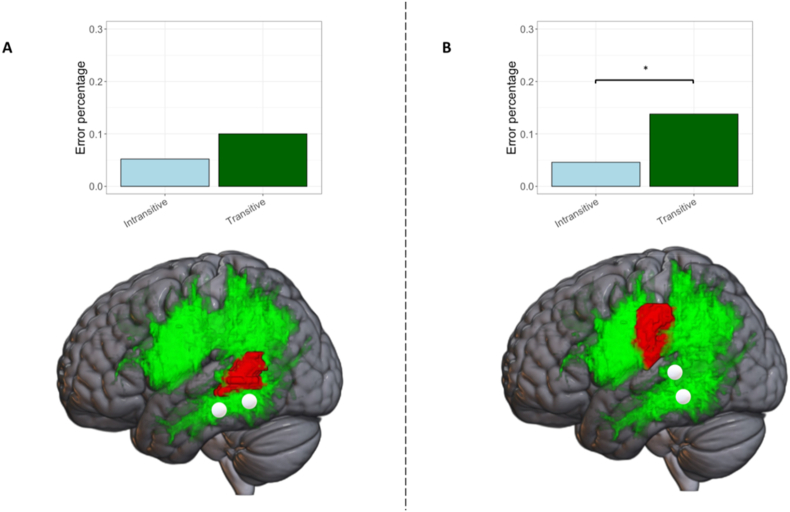
Results of cross-validation analysis. (A) nTMS-induced error percentages according to verb type for patients with tumors in left posterior temporal regions. (B) nTMS-induced error percentages according to verb type for patients with tumors that spare left posterior temporal areas. Red 3D masses represent exemplary tumors of 2 patients. (For interpretation of the references to color in this figure legend, the reader is referred to the Web version of this article.)

The aim of this analysis was to verify that potential effects of transitive verbs when stimulating the temporal terminations of the AF are exclusively present when tumors have not infiltrated or displaced the temporal terminations of the AF. Eight patients were identified as having tumors located in middle/posterior temporal areas. A Barnard's test between the nTMS-induced errors for transitive and intransitive verbs for the remaining 14 patients without posterior temporal tumors indicated that significantly more errors were induced during naming of transitive verbs (W = −2.11, p = 0.03). In contrast, the same effect did not emerge when examining the induced errors for the two verb types in the eight patients with posterior temporal tumors (W = −1.18, p = 0.24; see Fig. 6).

### 3.6 Error-type analysis

The most frequently induced error was hesitation on the target (37.5%), followed by semantic paraphasias (23.2%), anomias (16.1%), articulation errors (13.9%), no responses (3.6%), hesitations on the sentence (3.6%), phonological paraphasias (1.4%) and grammatical errors (0.7%). Looking at the stimulations of AF temporal terminations, Barnard's tests demonstrated that the only error type that differed more frequently induced during transitive rather than intransitive verb naming was semantic paraphasias (W = −2.02; p = 0.02). Table 1 shows percentages of induced errors for the AF temporal terminations according to verb type.

**Table 1:** Percentages of induced errors during stimulation of the AF temporal terminations according to verb and error type.

Error type	Verb type	Percentage
Hesitation	Transitive	5.1%
	Intransitive	2.4%
Anomia	Transitive	2.3%
	Intransitive	1.6%
**Semantic paraphasia**	**Transitive**	**3.3%**
	**Intransitive**	**0.0%**
Articulation error	Transitive	0.5%
	Intransitive	0.0%
Phonological paraphasia	Transitive	0.5%
	Intransitive	0.0%
Grammatical error	Transitive	0.0%
	Intransitive	0.0%

## 4 Discussion

The present study examined the influence on naming of transitive and intransitive verbs during presurgical nTMS language mapping in a group of 22 patients with gliomas in the left language-dominant hemisphere. During naming of transitive verbs, nTMS induced more language errors, especially when stimulating the temporal terminations of the AF and peritumoral regions of temporoparietal tumors. These results were cross-validated. The qualitative analysis demonstrated that nTMS induces more semantic paraphasias during transitive verb naming when stimulating the left posterior temporal cortex. These findings highlight the impact of linguistic stimuli on clinical language mappings of patients with brain tumors.

Higher error rates are only observed when patients name pictures of transitive verbs (10.02%) compared to intransitive verbs (6.7%). The reasons behind this higher error rate for transitive verbs can be found in linguistic theory and models of the neurobiological basis of language. Processing of verb-related linguistic variables has been recently argued to take place in posterior temporal regions (Matchin et al., 2020; Ouden et al., 2019). Considering that higher nTMS error rates during transitive verb naming specifically emerged when stimulating the temporal terminations of the AF, our findings verify that more complex semantic or syntactic processing seems to take place in posterior temporal regions. This renders transitive verbs particularly relevant for patients that undergo nTMS language mapping for tumors around the temporal terminations of the AF.

Interestingly, the cross-validation analysis showed that the increased error rate for transitive verbs is absent when tumors have displaced the temporal terminations of the AF. The reason behind this can be twofold:

First, it has been previously reported that the growth of gliomas induces functional reorganization of linguistic processes in the right hemisphere (Krieg et al., 2013; Piai et al., 2020). If indeed transitive verbs are processed more in left posterior temporal regions, glioma growth around this area might induce reorganization of functions that are relevant for transitive verb processing. For example, it has been demonstrated that during a semantic task MEG activity that typically appears in left frontal regions for healthy individuals, can be found in homologous right hemisphere regions for glioma patients (Piai et al., 2020). For our group of patients, this suggests that a potential effect for transitive verbs might be found when stimulating right hemisphere posterior temporal regions. Unfortunately, in this study we did not stimulate homologous areas in the right hemisphere. Hence, we cannot provide evidence for this point.

Second, the displacement of the temporal terminations of the AF may play a role. Language is currently thought to be the result of multiple network interactions rather than the mere outcome of a specific cortical region (Duffau et al., 2014). Following this definition, we can assume that, when processing a transitive verb, information needs to be transferred between regions that are connected by the AF. This point regarding information transfer between AF connected regions for the processing of more complex verbs is also supported by neurobiological models of verb processing (Matchin and Hickok, 2020; Thompson and Meltzer-Asscher, 2014). If tumor growth has caused displacement of AF endings, this could lead to the absence of the transitivity effect when stimulating posterior temporal regions. This is also evident in our cross-validation analysis (3.5), where for some of the patients the temporal stimulation points had to be placed in inferior temporal regions. However, this does not necessarily imply that these patients will be impaired in transitive action processing during baseline naming, as differences between transitive and intransitive verbs are rarely spotted based solely on accuracy measures (Thompson et al., 2010). This is supported by our data (see Supplementary Material).

The analysis of error types showed that when stimulating the left temporal terminations of the arcuate fasciculus, the error type that was primarily induced during transitive action naming were semantic paraphasias. Typically, patients with posterior temporal lesions present semantic deficits (Dronkers et al., 2004). Intraoperative stimulation has also demonstrated that DES over the posterior temporal lobe can induce semantic errors (Corina et al., 2010). By using items with a higher semantic load (i.e., with an *agent* + theme vs only an *agent*) in our naming tasks, such as transitive verbs, we increase the probability of identifying language areas and as a result, potentially minimizing iatrogenic damage more so than with other stimuli used in naming tasks (e.g., intransitive verbs).

It is important to note that although induced error rates differ between transitive and intransitive items, it is still uncertain whether statistical distinctions translate to clinical significance. This is a relevant point to the broader context of preoperative language mapping with nTMS (Picht et al., 2013; Sollmann et al., 2017; Tarapore et al., 2013). Considering that nTMS-based language mapping has been shown to enhance clinical procedures by reducing surgical duration and minimizing craniotomy size (Sollmann et al., 2015), we can assume that by identifying more language-positive points through the use of transitive verbs, we can establish a more conservative mapping. This could in turn prevent the removal of functionally relevant regions, particularly in cases where patients cannot undergo awake surgery. However, a definitive answer to this issue requires a clinical study encompassing both pre- and postoperative assessments for both verb types. This study could determine whether the preservation of preoperative positive points with transitive verbs is associated with better verb production outcomes.

Regarding limitations and future directions, the fiber tracking method used in the present study was DTI. It has been reported that DTI underperforms compared to other tractography methods, especially when it comes to certain neuropathologies, such as tumor infiltrated fiber reconstructions (Becker et al., 2022). Nevertheless, it should be noted that despite certain limitations most clinical software still relies on DTI algorithms (Becker et al., 2020). It should also be highlighted that tractography is by definition an estimation of the structure of the AF and results should be interpreted with caution (Ntemou et al., 2023; Reisch et al., 2022). Additionally, presurgical language mapping with nTMS might be increasingly used in research and clinical settings due to its advantages compared to fMRI, but concordance with the gold standard of DES mapping is still relatively low (Picht et al., 2013; Tarapore et al., 2013). As a next step, our findings should be validated by using not only different tractography algorithms or nTMS mappings in the right hemisphere, but also DES evidence, as this is the current gold standard for language mapping in this population. Our research group is currently working towards ways to validate the present findings and refine the methods. Lastly, it should be highlighted that the key error type induced more during transitive rather than intransitive naming was semantic paraphasias when stimulating posterior temporal terminations. Considering than temporal regions are densely populated also with ventral pathways, future work could contrast stimulations over the temporal terminations of the AF with temporal stimulations that do not overlap with AF terminations. This would establish whether induction of semantic paraphasias was indeed due to stimulation of temporal AF terminations or might have been a by-product of potential co-stimulation of other ventral pathways involved in semantic processing.

## 5 Conclusion

The present work demonstrates that, during presurgical language mapping of patients with gliomas, nTMS induces more errors during the naming of more complex verb types (i.e., transitives) as compared to less complex verbs (i.e., intransitives). The effect is particularly prominent when stimulating the temporal terminations of the left AF during transitive action naming and it points towards the suitability of more complex action stimuli for the mapping of left posterior temporal regions. Especially, the increased number of semantic errors during stimulation of temporal AF terminations with transitive actions, potentially indicates semantic processes that can be better identified when transitive actions are included in language mapping protocols.

## Declaration of competing interest

The authors declare that they have no known competing financial interests or personal relationships that could have appeared to influence the work reported in this paper.

## Data Availability

Data will be made available on request.

## References

[bib1] Barnard G.A. (1947). Significance tests for 2 × 2 tables. Biometrika.

[bib2] Bastiaanse R., Jonkers R. (1998). Verb retrieval in action naming and spontaneous speech in agrammatic and anomic aphasia. Aphasiology.

[bib3] Bates D., Mächler M., Bolker B., Walker S. (2014).

[bib4] Becker D., Neher P., Jungk C., Jesser J., Pflüger I., Brinster R., Bendszus M., Bruckner T., Maier-Hein K., Scherer M., Unterberg A. (2022). Comparison of diffusion signal models for fiber tractography in eloquent glioma surgery–determination of accuracy under awake craniotomy conditions. World Neurosurg..

[bib5] Becker D., Scherer M., Neher P., Jungk C., Jesser J., Pflüger I., Brinster R., Bendszus M., Bruckner T., Maier-Hein K., Unterberg A. (2020). Going beyond diffusion tensor imaging tractography in eloquent glioma surgery–high-resolution fiber tractography: Q-ball or constrained spherical deconvolution?. World Neurosurg..

[bib6] Catani M., de Schotten M.T. (2008). A diffusion tensor imaging tractography atlas for virtual in vivo dissections. Cortex.

[bib7] Corina D.P., Loudermilk B.C., Detwiler L., Martin R.F., Brinkley J.F., Ojemann G. (2010). Analysis of naming errors during cortical stimulation mapping: implications for models of language representation. Brain Lang..

[bib8] De Bleser R., Kauschke C. (2003). Acquisition and loss of nouns and verbs: parallel or divergent patterns?. J. Neurolinguistics.

[bib9] den Ouden D.B., Zhu M.W. (2022). Neuromodulation of verb-transitivity judgments. J. Neurolinguistics.

[bib10] den Ouden D.-B., Fix S.C., Parrish T.B., Thompson C.K. (2009). Argument structure effects in action verb naming in static and dynamic conditions. J. Neurolinguistics.

[bib11] Dronkers N.F., Wilkins D.P., Van Valin R.D., Redfern B.B., Jaeger J.J. (2004). Lesion analysis of the brain areas involved in language comprehension. Cognition.

[bib12] Duffau H., Moritz-Gasser S., Mandonnet E. (2014). A re-examination of neural basis of language processing: proposal of a dynamic hodotopical model from data provided by brain stimulation mapping during picture naming. Brain Lang..

[bib13] Erguler K. (2016). Barnard: Barnard's Unconditional Test. R Package Version 1.8. [Computer Software]. https://CRAN.R-project.org/package=Barnard.

[bib14] Fekonja L., Wang Z., Bährend I., Rosenstock T., Rösler J., Wallmeroth L., Vajkoczy P., Picht T. (2019). Manual for clinical language tractography. Acta Neurochir..

[bib15] Finocchiaro C., Capasso R., Cattaneo L., Zuanazzi A., Miceli G. (2015). Thematic role assignment in the posterior parietal cortex: a TMS study. Neuropsychologia.

[bib16] Grimshaw J. (1990).

[bib17] Hernandez-Pavon J.C., Mäkelä N., Lehtinen H., Lioumis P., Mäkelä J.P. (2014). Effects of navigated TMS on object and action naming. Front. Hum. Neurosci..

[bib18] Jenkinson M., Beckmann C.F., Behrens T.E., Woolrich M.W., Smith S.M. (2012). FSL. Neuroimage.

[bib19] Krieg S.M., Lioumis P., Mäkelä J.P., Wilenius J., Karhu J., Hannula H., Savolainen P., Lucas C.W., Seidel K., Laakso A., Islam M., Vaalto S., Lehtinen H., Vitikainen A.-M., Tarapore P.E., Picht T. (2017). Protocol for motor and language mapping by navigated TMS in patients and healthy volunteers; workshop report. Acta Neurochir..

[bib20] Krieg S.M., Sollmann N., Hauck T., Ille S., Foerschler A., Meyer B., Ringel F. (2013). Functional language shift to the right hemisphere in patients with language-eloquent brain tumors. PLoS One.

[bib21] Lutkenhoff E.S., Rosenberg M., Chiang J., Zhang K., Pickard J.D., Owen A.M., Monti M.M. (2014). Optimized brain extraction for pathological brains (optiBET). PLoS One.

[bib22] Makris N., Goldstein J.M., Kennedy D., Hodge S.M., Caviness V.S., Faraone S.V., Tsuang M.T., Seidman L.J. (2006). Decreased volume of left and total anterior insular lobule in schizophrenia. Schizophr. Res..

[bib23] Malyutina S., den Ouden D.-B. (2017). Task-dependent neural and behavioral effects of verb argument structure features. Brain Lang..

[bib24] Matchin W., Basilakos A., Stark B.C., den Ouden D.-B., Fridriksson J., Hickok G. (2020). Agrammatism and paragrammatism: a cortical double dissociation revealed by lesion-symptom mapping. Neurobiol. Lang..

[bib25] Matchin W., Hickok G. (2020). The cortical organization of syntax. Cerebr. Cortex.

[bib26] Ntemou E., Ohlerth A.-K., Ille S., Krieg S.M., Bastiaanse R., Rofes A. (2021). Mapping verb retrieval with nTMS: the role of transitivity. Front. Hum. Neurosci..

[bib27] Ntemou E., Svaldi C., Jonkers R., Picht T., Rofes A. (2023). Verb and sentence processing with TMS: a systematic review and meta-analysis. Cortex.

[bib28] Ohlerth A.-K., Bastiaanse R., Negwer C., Sollmann N., Schramm S., Schröder A., Krieg S.M. (2021). Bihemispheric navigated transcranial magnetic stimulation mapping for action naming compared to object naming in sentence context. Brain Sci..

[bib29] Ohlerth A.-K., Valentin A., Vergani F., Ashkan K., Bastiaanse R. (2020). The verb and noun test for peri-operative testing (VAN-POP): standardized language tests for navigated transcranial magnetic stimulation and direct electrical stimulation. Acta Neurochir..

[bib30] Oldfield R.C. (1971). The assessment and analysis of handedness: the Edinburgh inventory. Neuropsychologia.

[bib31] Ouden D., Malyutina S., Basilakos A., Bonilha L., Gleichgerrcht E., Yourganov G., Hillis A.E., Hickok G., Rorden C., Fridriksson J. (2019). Cortical and structural‐connectivity damage correlated with impaired syntactic processing in aphasia. Hum. Brain Mapp..

[bib32] Piai V., De Witte E., Sierpowska J., Zheng X., Hinkley L.B., Mizuiri D., Knight R.T., Berger M.S., Nagarajan S.S. (2020). Language neuroplasticity in brain tumor patients revealed by magnetoencephalography. J. Cognit. Neurosci..

[bib33] Picht T., Krieg S.M., Sollmann N., Rösler J., Niraula B., Neuvonen T., Savolainen P., Lioumis P., Mäkelä J.P., Deletis V., Meyer B., Vajkoczy P., Ringel F. (2013). A comparison of language mapping by preoperative navigated transcranial magnetic stimulation and direct cortical stimulation during awake surgery. Neurosurgery.

[bib34] R Core Team (2020). R: A Language and Environment for Statistical Computing. https://www.R-project.org/.

[bib35] Rappaport Hovav M., Levin B. (2002). Change of state verbs: implications for theories of argument projection. Annu. Meeting Berkeley Linguistics Soc..

[bib36] Reisch K., Böttcher F., Tuncer M.S., Schneider H., Vajkoczy P., Picht T., Fekonja L.S. (2022). Tractography-based navigated TMS language mapping protocol. Front. Oncol..

[bib37] Rofes A., Mahon B.Z., Mandonnet E., Herbet G. (2021). Intraoperative Mapping of Cognitive Networks: Which Tasks for Which Locations.

[bib38] Rofes A., Miceli G. (2014). Language mapping with verbs and sentences in awake surgery: a review. Neuropsychol. Rev..

[bib39] Rofes A., Spena G., Talacchi A., Santini B., Miozzo A., Miceli G. (2017). Mapping nouns and finite verbs in left hemisphere tumors: a direct electrical stimulation study. Neurocase.

[bib40] Shetreet E., Palti D., Friedmann N., Hadar U. (2007). Cortical representation of verb processing in sentence comprehension: number of complements, subcategorization, and thematic frames. Cerebr. Cortex.

[bib41] Smith S.M. (2002). Fast robust automated brain extraction. Hum. Brain Mapp..

[bib42] Sollmann N., Ille S., Hauck T., Maurer S., Negwer C., Zimmer C., Ringel F., Meyer B., Krieg S.M. (2015). The impact of preoperative language mapping by repetitive navigated transcranial magnetic stimulation on the clinical course of brain tumor patients. BMC Cancer.

[bib43] Sollmann N., Meyer B., Krieg S.M. (2017). Implementing functional preoperative mapping in the clinical routine of a neurosurgical department: technical note. World Neurosurg..

[bib44] Suissa S., Shuster J.J. (1985). Exact unconditional sample sizes for the 2 × 2 binomial trial. J. Roy. Stat. Soc..

[bib45] Tarapore P.E., Findlay A.M., Honma S.M., Mizuiri D., Houde J.F., Berger M.S., Nagarajan S.S. (2013). Language mapping with navigated repetitive TMS: proof of technique and validation. Neuroimage.

[bib46] Thompson C.K., Bonakdarpour B., Fix S.F. (2010). Neural mechanisms of verb argument structure processing in agrammatic aphasic and healthy age-matched listeners. J. Cognit. Neurosci..

[bib47] Thompson C., Meltzer-Asscher A., Bachrach A., Roy I., Stockall L. (2014).

[bib48] Tuncer M.S., Salvati L.F., Grittner U., Hardt J., Schilling R., Bährend I., Silva L.L., Fekonja L.S., Faust K., Vajkoczy P., Rosenstock T., Picht T. (2021). Towards a tractography-based risk stratification model for language area associated gliomas. Neuroimage: Clin..

[bib49] Vercesi L., Sabnis P., Finocchiaro C., Cattaneo L., Tonolli E., Miceli G. (2020). The role of the l-IPS in the comprehension of reversible and irreversible sentences: an rTMS study. Brain Struct. Funct..

[bib50] Ward E., Brownsett S.L.E., McMahon K.L., Hartwigsen G., Mascelloni M., de Zubicaray G.I. (2022). Online transcranial magnetic stimulation reveals differential effects of transitivity in left inferior parietal cortex but not premotor cortex during action naming. Neuropsychologia.

