# Supplementary material for: Mapping action naming in patients with gliomas: The influence of transitivity
Source: Neuroimage Rep. 2023 Sep 21;3(4):100184. doi: 10.1016/j.ynirp.2023.100184 (PMC12172801; doi:10.1016/j.ynirp.2023.100184)
Supplement: Multimedia component 1 [file mmc1.docx]

# **Supplementary Material**

**Table S1:** Results of the logistic model for error binary as a function of verb type with random intercepts for item and participant.

|  |  |  | **Error binary** |  |  |
| --- | --- | --- | --- | --- | --- |
| **Predictors** |  | *Odds Ratios* | *CI* | *p* |  |
| (Intercept) |  | 0.05 | 0.03 – 0.08 | <0.001 |  |
| Verb type [Transitive] |  | 1.84 | 1.11 – 3.07 | **0.019** |  |
| **Random Effects** |  |  |  |  |  |
| σ2 | 3.29 |  |  |  |  |
| τ_00_ Item | 0.67 |  |  |  |  |
| τ_00_ Participant | 0.18 |  |  |  |  |
| ICC | 0.20 |  |  |  |  |
| N Item | 75 |  |  |  |  |
| N Participant | 22 |  |  |  |  |
| Observations | 3106 |  |  |  |  |
| Marginal R2 / Conditional R2 | 0.020 / 0.221 |  |  |  |  |

**Table S2:** Differences between transitive and intransitive verbs at baseline.

| **Patient** | **Error rate - transitives** | **Error rate - intransitives** | **Chi-square (p value)** |
| --- | --- | --- | --- |
| P01 | 0.557692308 | 0.545454545 | 0.930 |
| P02 | 0.384615385 | 0.227272727 | 0.19 |
| P03 | 0.461538462 | 0.363636364 | 0.619 |
| P04 | 0.365384615 | 0.136363636 | 0.123 |
| P05 | 0.326923077 | 0.454545455 | 0.511 |
| P06 | 0.461538462 | 0.272727273 | 0.463 |
| P07 | 0.307692308 | 0.19047619 | 0.382 |
| P08 | 0.538461538 | 0.454545455 | 0.817 |
| P09 | 0.384615385 | 0.318181818 | 0.736 |
| P10 | 0.269230769 | 0.227272727 | 0.705 |
| P11 | 0.461538462 | 0.363636364 | 0.341 |
| P12 | 0.384615385 | 0.227272727 | 0.274 |
| P13 | 0.230769231 | 0.272727273 | 0.964 |
| P14 | 0.346153846 | 0.272727273 | 0.578 |
| P15 | 0.346153846 | 0.272727273 | 0.578 |
| P16 | 0.192307692 | 0.136363636 | 0.78 |
| P17 | 0.307692308 | 0.136363636 | 0.195 |
| P18 | 0.192307692 | 0.136363636 | 0.442 |
| P19 | 0.230769231 | 0.318181818 | 0.652 |
| P20 | 0.019230769 | 0.090909091 | 0.443 |
| P21 | 0.307692308 | 0.136363636 | 0.195 |
| P22 | 0.230769231 | 0.090909091 | 0.106 |

**Table S3:** Stimulation targets in MNI space. Coordinates are given in mm.

| **Patient** | **MNI_coordinates** |
| --- | --- |
| P01 | -48.6736  31.4645  3.17068 |
| P01 | -52.6253  14.5602  19.2167 |
| P01 | -59.8502  -37.6987  37.0932 |
| P01 | -39.9266  -69.9176  39.0365 |
| P01 | -55.2334  -53.2033  -4.69244 |
| P01 | -55.8278  -42.3111  -13.7612 |
| P01 | -61.9062  -27.5964  19.1689 |
| P01 | -62.1744  -35.0188  17.2614 |
| P01 | -61.0322  -41.1493  13.906 |
| P01 | -59.4318  -44.5956  6.54746 |
| P01 | -59.6872  -37.7726  3.14844 |
| P01 | -59.3938  -31.6901  0.480726 |
| P01 | -59.9133  -24.7548  3.44124 |
| P01 | -61.319  -22.9921  11.1822 |
| P01 | -61.8696  -31.9555  10.4138 |
| P01 | -57.8445  -31.8691  -7.17495 |
| P02 | -60.4505  6.31041  18.1608 |
| P02 | -53.8203  30.813  10.8707 |
| P02 | -45.9504  -48.2458  46.4709 |
| P02 | -36.5587  -70.6328  41.3529 |
| P02 | -58.2902  -32.7022  -14.2438 |
| P02 | -59.9122  -37.1523  7.26882 |
| P02 | -48.3775  -55.0708  34.8273 |
| P02 | -49.1013  -58.0244  28.2603 |
| P02 | -51.1102  -57.1705  21.2354 |
| P02 | -54.3762  -50.2569  19.9521 |
| P02 | -57.3453  -43.1443  18.0528 |
| P02 | -56.6719  -40.9153  25.7303 |
| P02 | -54.0349  -45.7793  29.103 |
| P02 | -51.1377  -49.5781  33.5173 |
| P02 | -52.0884  -52.3162  26.5467 |
| P02 | -54.8646  -46.6732  24.5179 |
| P03 | -53.1008  9.37002  12.9286 |
| P03 | -55.9487  -10.09  20.9599 |
| P03 | -44.6648  -38.5033  43.3687 |
| P03 | -44.5521  -64.7035  32.3015 |
| P03 | -57.4912  -33.6414  5.49019 |
| P03 | -57.2821  -44.411  -2.62973 |
| P03 | not possible due to technical issues |
| P03 | -56.786  -12.4924  1.70355 |
| P03 | -57.3428  -22.6971  5.06393 |
| P03 | -48.543  -3.48427  34.8493 |
| P03 | -48.8708  13.4493  23.6642 |
| P03 | -54.5168  -20.5683  29.8793 |
| P03 | -54.796  -39.7241  28.4655 |
| P03 | not possible due to technical issues |
| P03 | -54.7408  -60.1587  12.9754 |
| P03 | -49.2299  -17.144  37.8701 |
| P04 | -49.7405  25.9003  15.8892 |
| P04 | -51.5509  3.49113  26.0959 |
| P04 | -54.8612  -46.9338  40.3612 |
| P04 | -47.5864  -67.5215  34.958 |
| P04 | -59.1925  -44.5279  11.1678 |
| P04 | -57.7641  -37.2478  -6.85553 |
| P04 | -54.7821  -4.78872  15.9452 |
| P04 | -56.2842  -10.4269  6.99317 |
| P04 | -56.6923  -8.99299  -0.949201 |
| P04 | -52.5108  11.3981  19.6221 |
| P04 | -55.3448  15.5161  7.36173 |
| P04 | -58.1135  13.3098  -2.76409 |
| P04 | -58.3034  2.71586  -6.19784 |
| P04 | -54.607  -6.44009  25.617 |
| P04 | -55.4886  3.97931  13.8174 |
| P04 | -36.215  3.91533  4.25387 |
| P05 | -49.5394  25.6379  22.6095 |
| P05 | -45.2499  22.2025  37.3233 |
| P05 | -60.9319  -39.5028  9.17961 |
| P05 | -61.9706  -25.8916  -6.49069 |
| P05 | -53.92  -50.2964  39.9296 |
| P05 | -43.3195  -57.8199  48.4709 |
| P05 | -48.8306  -20.9139  50.0598 |
| P05 | -50.7728  -36.1023  46.9792 |
| P05 | -58.6804  -34.3754  32.0299 |
| P05 | -53.0676  -11.1543  42.1654 |
| P05 | -60.0432  -25.4096  22.943 |
| P05 | -59.5571  -15.0778  24.3649 |
| P05 | -57.4788  -8.24452  32.1707 |
| P05 | -42.4927  -43.2861  54.1006 |
| P05 | -54.1844  -23.6236  38.8789 |
| P05 | -55.4657  -28.7636  41.0692 |
| P06 | -52.2005  23.8894  13.7475 |
| P06 | -45.5597  22.77  26.7701 |
| P06 | -62.0986  -25.8208  0.681477 |
| P06 | -62.8061  -27.4542  -8.3732 |
| P06 | -62.0904  -30.1586  36.7075 |
| P06 | -58.4757  -43.6236  40.6688 |
| P06 | -53.4851  12.3867  24.888 |
| P06 | -56.3499  3.24522  27.7225 |
| P06 | -60.1523  -2.20918  23.3704 |
| P06 | -62.5326  -4.26882  15.7743 |
| P06 | -62.8712  -2.57071  7.91689 |
| P06 | -62.5377  3.36785  0.40886 |
| P06 | -60.9459  11.3868  7.18456 |
| P06 | -57.5823  13.4205  16.3098 |
| P06 | -59.0981  5.87085  20.4035 |
| P06 | -61.8015  4.53171  12.5074 |
| P07 | -43.4649  33.1817  18.4046 |
| P07 | -53.4477  11.7844  20.3804 |
| P07 | -60.3059  -37.3564  15.4856 |
| P07 | -59.7284  -43.7846  3.22654 |
| P07 | -40.5685  -57.8506  56.1165 |
| P07 | -48.7041  -29.754  50.4026 |
| P07 | -57.1778  -16.5593  1.91264 |
| P07 | -55.2867  -9.61603  -8.27887 |
| P07 | -57.868  -28.6494  5.7515 |
| P07 | -56.917  -37.0509  -5.06315 |
| P07 | -56.5705  -33.9186  -13.5897 |
| P07 | -56.0989  -26.7602  -19.0027 |
| P07 | -54.5553  -15.1831  -17.2388 |
| P07 | -57.2355  -27.0193  -2.71153 |
| P07 | -56.1404  -19.8376  -9.29383 |
| P07 | -57.6743  -23.973  12.1346 |
| P08 | -39.2563 30.648 16.0101 |
| P08 | -55.0657 5.7137 16.6877 |
| P08 | -44.3902 -57.1949 41.1532 |
| P08 | -36.6706 -66.9848 38.0811 |
| P08 | -56.594 -39.1879 -17.9995 |
| P08 | -57.7097 -22.9323 -18.9968 |
| P08 | -58.4905 1.54996 -1.04703 |
| P08 | -58.1509 -2.42295 -10.7241 |
| P08 | -57.0158 -1.04431 -19.7075 |
| P08 | -56.6718 6.29555 -22.7847 |
| P08 | -53.5047 16.0244 -27.0379 |
| P08 | -48.6908 22.875 -22.7371 |
| P08 | -49.5578 22.0933 -11.6792 |
| P08 | -54.3543 14.2954 -3.13525 |
| P08 | -55.1927 12.9606 -10.2583 |
| P08 | -55.6216 12.194 -17.2959 |
| P09 | -47.8844  18.6801  21.1785 |
| P09 | -43.1676  27.2868  25.6374 |
| P09 | -55.8179  -33.4978  12.0459 |
| P09 | -55.8178  -39.8011  -4.46226 |
| P09 | -50.3203  -31.4079  43.8847 |
| P09 | -49.3774  -58.996  33.5808 |
| P09 | -53.1574  -21.1881  -1.52849 |
| P09 | -54.4679  -26.3998  -2.42675 |
| P09 | -54.4856  -31.7942  -11.0394 |
| P09 | -52.7413  -12.6757  7.20375 |
| P09 | -52.8976  -6.39649  -0.478716 |
| P09 | -54.1906  -24.1103  -16.2189 |
| P09 | -53.1522  -14.8041  -1.70148 |
| P09 | -53.5478  -19.0309  -9.68165 |
| P09 | -53.6827  -7.87391  -7.67745 |
| P09 | -53.6922  -15.5813  -13.8017 |
| P10 | -49.0636 25.2424 17.4241 |
| P10 | -54.9311 -0.776721 29.2195 |
| P10 | -59.8762 -42.7151 8.40236 |
| P10 | -60.3852 -38.5465 -3.33969 |
| P10 | -52.8338 -54.078 33.9248 |
| P10 | -59.1424 -38.3769 30.4316 |
| P10 | -55.6423 0.920403 13.9078 |
| P10 | -51.9139 14.7748 10.4957 |
| P10 | -47.8077 26.9453 6.23139 |
| P10 | -41.7606 34.0418 17.0617 |
| P10 | -36.0522 20.3981 32.7976 |
| P10 | -48.2468 5.75538 32.3267 |
| P10 | -50.3775 14.7456 20.524 |
| P10 | -58.1555 -9.98974 6.41573 |
| P10 | -54.9496 2.71125 -2.96211 |
| P10 | not possible due to technical issues |
| P11 | -59.3223  -9.01307  22.7948 |
| P11 | -59.6636  -19.464  27.1883 |
| P11 | -52.5286  -53.5052  40.9691 |
| P11 | -39.6664  -70.8402  44.4545 |
| P11 | -59.0205  -39.4692  11.3958 |
| P11 | -60.1749  -39.6153  -3.17202 |
| P11 | -47.8203  -0.19561  42.9176 |
| P11 | -42.4667  11.0761  44.5381 |
| P11 | -54.837  -1.67778  30.935 |
| P11 | -57.7193  0.506904  18.0959 |
| P11 | -56.4978  2.77766  12.5021 |
| P11 | -54.9255  23.7781  4.51125 |
| P11 | -49.3043  28.6545  20.3633 |
| P11 | -45.3319  20.5134  35.8411 |
| P11 | -53.115  18.5314  20.4852 |
| P11 | -51.9701  9.64481  31.3468 |
| P12 | -51.1804  20.7584  14.888 |
| P12 | -55.4917  6.81397  19.2089 |
| P12 | -61.0182  -45.4282  8.55867 |
| P12 | -60.7457  -44.8323  -1.80499 |
| P12 | -54.1702  -49.6791  38.1523 |
| P12 | -47.3933  -52.74  45.7852 |
| P12 | -62.5135  -23.5158  10.6913 |
| P12 | -62.6387  -34.2226  14.7129 |
| P12 | -62.1397  -25.6597  3.12702 |
| P12 | -61.6037  -32.2341  -1.18079 |
| P12 | -62.5325  -31.7064  7.90335 |
| P12 | -61.8749  -38.768  4.9783 |
| P12 | -61.3142  -42.5972  16.0058 |
| P12 | -62.7389  -28.3825  18.9474 |
| P12 | -61.2875  -26.8797  -5.06428 |
| P12 | -61.8496  -38.9749  18.6479 |
| P13 | -48.345  25.1538  14.671 |
| P13 | -55.6026  10.8367  8.12108 |
| P13 | -54.932  -39.158  31.2307 |
| P13 | -48.5052  -52.5401  37.9498 |
| P13 | -57.7821  -32.6255  -1.79224 |
| P13 | -57.5495  -27.7241  -13.3292 |
| P13 | -55.5307  -51.6923  15.4556 |
| P13 | -49.0017  -65.6895  17.4447 |
| P13 | -40.9571  -68.741  31.5798 |
| P13 | -33.2879  -66.418  41.7008 |
| P13 | -30.3616  -57.6183  51.1334 |
| P13 | -39.6057  -44.1701  49.2505 |
| P13 | -49.6275  -36.4893  37.5169 |
| P13 | -55.1161  -39.6782  19.2051 |
| P13 | -50.4742  -50.5199  29.2714 |
| P13 | -39.604  -54.7287  43.7296 |
| P14 | -46.5371  21.6113  7.17348 |
| P14 | -50.1394  6.23694  19.9012 |
| P14 | -45.8444  -25.1006  50.7018 |
| P14 | -41.1041  -32.2847  56.6768 |
| P14 | -55.1373  -37.9869  24.2026 |
| P14 | -51.7061  -49.2872  4.18089 |
| P14 | -55.7464  -21.8506  16.832 |
| P14 | -55.88  -30.2882  15.7007 |
| P14 | -55.0862  -37.9256  11.6904 |
| P14 | -53.145  -38.2255  1.80619 |
| P14 | -52.3384  -33.0975  -5.9346 |
| P14 | -53.2884  -23.3167  -4.34384 |
| P14 | -54.1203  -16.973  3.52097 |
| P14 | -54.8553  -17.7076  9.08398 |
| P14 | not possible due to technical issues |
| P14 | -54.2253  -31.8063  4.69351 |
| P15 | -51.7805  -1.43045  29.395 |
| P15 | -46.6285  -4.21816  40.7778 |
| P15 | -43.9687  -32.9053  51.2763 |
| P15 | -48.5668  -44.3307  42.1757 |
| P15 | -55.3734  -23.9525  19.3333 |
| P15 | -55.0568  -24.0131  8.69286 |
| P15 | -54.4519  -5.484  18.4718 |
| P15 | -55.0624  -13.2576  17.3199 |
| P15 | -54.5917  -5.37523  10.6575 |
| P15 | -53.215  10.052  19.1236 |
| P15 | -54.6063  6.68172  8.6677 |
| P15 | -43.5056  29.1577  29.604 |
| P15 | -32.6005  46.1027  27.8156 |
| P15 | -11.4062  56.8134  28.1591 |
| P15 | -51.7716  -15.5219  32.5743 |
| P15 | -42.0595  5.45933  42.2392 |
| P16 | -47.7292  14.6097  24.7573 |
| P16 | -51.3861  -1.31815  28.0832 |
| P16 | -53.2992  -45.6486  28.7335 |
| P16 | -43.9369  -60.3324  36.4525 |
| P16 | -53.7205  -48.1728  12.5755 |
| P16 | -53.0695  -37.0041  -8.29669 |
| P16 | -28.1304  -11.4709  60.2553 |
| P16 | -36.6591  -0.219886  48.6672 |
| P16 | -12.0389  -4.31576  68.498 |
| P16 | -21.8466  9.1625  58.652 |
| P16 | -20.1328  -22.564  67.8561 |
| P16 | -41.5066  -28.0253  49.3019 |
| P16 | -44.8734  4.89833  36.1754 |
| P16 | -33.8356  25.3147  39.3413 |
| P16 | -32.9797  -31.8501  58.1165 |
| P16 | -47.0191  -17.8929  41.4317 |
| P17 | -47.0166  17.6638  18.7115 |
| P17 | -49.8097  3.71856  23.7107 |
| P17 | -55.302  -24.0973  -7.05212 |
| P17 | -54.8157  -19.8097  5.32475 |
| P17 | -50.7721  -28.5389  26.5256 |
| P17 | -42.6375  -40.6266  37.4499 |
| P17 | -20.3271  -47.1844  58.9825 |
| P17 | -10.5993  -38.5389  63.7105 |
| P17 | -11.3694  -27.0102  63.5005 |
| P17 | -20.7578  -18.8405  58.5985 |
| P17 | -32.398  -16.7931  50.3711 |
| P17 | -37.905  -26.2269  45.2581 |
| P17 | -35.2075  -40.9701  45.7547 |
| P17 | -29.5184  -46.4071  51.2165 |
| P17 | -20.1811  -32.1522  58.0808 |
| P17 | -13.0164  -46.5715  63.0722 |
| P18 | -54.4808  18.1377  8.36239 |
| P18 | -56.0341  3.38039  22.4862 |
| P18 | -53.8447  -34.5302  36.6757 |
| P18 | -47.3697  -51.6319  40.6766 |
| P18 | -59.6566  -23.9006  3.00176 |
| P18 | -58.2755  -24.9636  -12.0169 |
| P18 | -46.0928  -64.8367  27.728 |
| P18 | -52.1626  -57.9193  22.2725 |
| P18 | -53.7096  -58.0832  14.3128 |
| P18 | -53.5559  -62.3499  7.71824 |
| P18 | -49.5301  -69.1161  5.54564 |
| P18 | -45.7018  -72.0535  14.3782 |
| P18 | -44.189  -71.0282  22.6282 |
| P18 | -50.4959  -64.6087  12.7028 |
| P18 | -49.6171  -67.3889  12.7688 |
| P18 | -55.8946  -52.3241  13.8709 |
| P19 | -44.2701  36.8958  16.5408 |
| P19 | -49.0145  25.8889  16.9347 |
| P19 | -56.3708  -53.1351  -0.253615 |
| P19 | -57.5949  -32.1804  -16.981 |
| P19 | -51.3909  -35.4654  42.916 |
| P19 | -52.8604  -49.2458  35.7549 |
| P19 | -59.0878  -21.1192  12.63 |
| P19 | -59.3659  -33.1104  15.2007 |
| P19 | -58.7545  -41.466  10.4407 |
| P19 | -58.0311  -43.0733  3.31578 |
| P19 | -57.7832  -36.5721  -9.27235 |
| P19 | -58.2792  -23.1343  -7.44936 |
| P19 | -58.2874  -17.7967  -0.617136 |
| P19 | -59.2702  -32.5397  8.84192 |
| P19 | -58.5265  -31.8526  -1.11817 |
| P19 | -58.2052  -13.6234  7.92236 |
| P20 | -53.3251  1.81612  20.851 |
| P20 | -52.1248  12.3259  16.3439 |
| P20 | -56.3047  -25.9787  26.4323 |
| P20 | -53.7983  -35.3657  34.5386 |
| P20 | -54.3211  -30.1714  -8.12369 |
| P20 | -55.5083  -32.8824  -0.87802 |
| P20 | -16.3585  -24.1483  66.1079 |
| P20 | -26.4227  -23.3637  59.5832 |
| P20 | -33.4061  -29.1921  55.4362 |
| P20 | -40.3086  -34.4761  50.2674 |
| P20 | -41.3206  -41.94  48.9049 |
| P20 | -40.1586  -49.9277  48.1571 |
| P20 | not possible due to technical issues |
| P20 | not possible due to technical issues |
| P20 | not possible due to technical issues |
| P20 | -22.429  -46.5763  62.6664 |
| P21 | not possible due to technical issues |
| P21 | not possible due to technical issues |
| P21 | not possible due to technical issues |
| P21 | not possible due to technical issues |
| P21 | not possible due to technical issues |
| P21 | not possible due to technical issues |
| P21 | -45.2664  -30.5416  55.7151 |
| P21 | -55.7578  -26.4584  42.6263 |
| P21 | -46.434  -9.31942  46.6113 |
| P21 | -54.0138  -41.6057  46.8839 |
| P21 | -59.8293  -37.1474  36.2658 |
| P21 | -62.0107  -29.366  23.6183 |
| P21 | -56.7667  -12.8126  33.0439 |
| P21 | -55.2818  0.366058  24.3787 |
| P21 | -58.1896  -4.67723  16.0606 |
| P21 | -61.1165  -15.7744  7.00899 |
| P22 | -45.0434  12.1929  13.2001 |
| P22 | -49.0774  1.3234  21.7814 |
| P22 | -51.6721  -33.5339  35.1439 |
| P22 | -42.8349  -50.5609  41.1687 |
| P22 | -54.0578  -25.9828  2.01376 |
| P22 | -54.0211  -30.1671  -9.42544 |
| P22 | -50.9834  -8.33441  11.7188 |
| P22 | -53.1756  -18.9248  8.04778 |
| P22 | -55.2074  -34.008  1.02924 |
| P22 | -53.3792  -29.6303  -18.7122 |
| P22 | -52.9574  -23.3972  -15.2501 |
| P22 | -48.2453  1.1305  8.52512 |
| P22 | -51.2817  -14.437  -2.63379 |
| P22 | -45.8713  10.177  23.6632 |
| P22 | not possible due to pain |
| P22 | not possible due to pain |

**Table S4:** T-test results for psycholinguistic properties between the two verb lists.

| Psycholinguistic variable | T - value | P - value |
| --- | --- | --- |
| Frequency | -1.36 | 0.17 |
| Age of acquisition | 0.331 | 0.74 |
| Naming accuracy | -0.277 | 0.78 |
